# Nerve Growth Factor and Brain-Derived Neurotrophic Factor in COVID-19

**DOI:** 10.3390/biology13110907

**Published:** 2024-11-07

**Authors:** Carla Petrella, Giampiero Ferraguti, Luigi Tarani, Francesca Tarani, Marisa Patrizia Messina, Marco Fiore

**Affiliations:** 1 Institute of Biochemistry and Cell Biology (IBBC-CNR), Via E. Ramarini, 32, Monterotondo Scalo, 00015 Rome, Italy; 2 Department of Experimental Medicine, Sapienza University of Rome, 00161 Rome, Italy; giampiero.ferraguti@uniroma1.it; 3 Department of Maternal Infantile and Urological Sciences, Sapienza University of Rome, 00185 Rome, Italy; luigi.tarani@uniroma1.it (L.T.); francesca.tarani@uniroma1.it (F.T.); marisapatrizia.messina@uniroma1.it (M.P.M.)

**Keywords:** nerve growth factor, brain-derived neurotrophic factor, SARS-CoV-2, COVID-19, acute symptomatology, long-COVID-19, pregnancy

## Abstract

Neurotrophins (NTs) constitute a family of small protein messengers that play a fundamental role in both the central and peripheral nervous systems. In particular, the nerve growth factor (NGF) and the brain-derived neurotrophic factor (BDNF) play a subtle role in the survival, differentiation, and functioning of neuronal populations, as well as in the fine regulation of immune functions. The SARS-CoV-2 infection was characterized by a sequela of symptoms (serious respiratory pathology, inflammatory storm, neurological discomfort, up to the less serious flu-like symptoms), which caused, at the end of 2023, more than 7 million deaths worldwide. Despite the official end of the pandemic, the physical and psychological consequences are currently the object of scientific research, both acute and chronic/long-lasting (Long-COVID-19). Given the multifactorial nature of the outcomes of SARS-CoV-2 infection in adults and children, several studies have investigated the potential involvement of the NGF and BDNF systems in the pathology. This narrative review aims to summarize the most recent evidence on this crucial topic.

## Simple Summary

This review explores recent findings on the role of neurotrophins, particularly NGF and BDNF, in the neurological and immune-related consequences of SARS-CoV-2 infection and its long-term outcomes.

## 1. Neurotrophins and Their Receptors

In the early 1950s, a key discovery by Rita Levi-Montalcini and Stanley Cohen essentially reshaped our neurobiology understanding. Their studies, originally concentrated on the mouse nervous system and the cells of a specific type of tumor known as Sarcoma 180, led to the discovery of a new class of biomolecules that played crucial roles in the development and function of neurons. They discovered that the tumor cells synthesized and released a distinctive protein, later named “nerve growth factor” (NGF), which was responsible for eliciting the maintenance and differentiation of both sympathetic and sensory neurons [1,2]. This was a pioneering discovery at the time, as it offered the first clear indication that proteins released from cells could induce the survival and growth of certain neuronal populations. NGF was later documented as the prototype of a wider family of neurotrophic factors, now recognized as neurotrophins (NTs), which would become a pivotal point for decades of studies aimed at understanding neuronal plasticity and development [3,4,5,6].

The detection of NGF encouraged widespread research efforts to disclose other proteins with similar properties. In the early 1980s, Barde and colleagues made a significant impact on the field by isolating from the pig brain the “brain-derived neurotrophic factor” (BDNF) [7,8,9]. BDNF, which has a high degree of sequence homology with NGF, was discovered to be another main cause involved in the neurons’ differentiation and survival [10,11]. This was shortly followed by the detection of several other NTs, including neurotrophin 3 (NT-3) and neurotrophin 4/5 (NT-4/5), both of which displayed similar structural and functional features [12,13,14]. Together, these novelties hardened the NT family as crucial players in the regulation of the nervous system’s neurons’ function and survival.

The genes encoding NTs are highly conserved among mammals and code for precursor proteins known as pre-NTs. These precursors undergo proteolytic processing to generate mature NTs, which are approximately 13 kDa in size and typically are non-covalently linked homodimers. One of the prominent characteristics of NTs is their simple isoelectric points, an uncommon peculiarity for secreted proteins [15,16,17]. This feature seems to play a key role in limiting their action, letting them exert limited effects within specific regions of the nervous system [18]. Another characteristic of the NT’s structure is the presence of a distinctive motif recognized as the “cysteine knot”, made by disulfide bridges. This configuration has been disclosed in other secreted proteins as well, such as platelet-derived growth factor (PDGF) and transforming growth factor-βs (TGF-βs), which indicates that it may be a well-conserved hallmark among peptides involved in intercellular signaling [19]. Intriguingly, the preservation of structural NTs goes beyond their cysteine knot motif. Indeed, with the exclusion of NT-4/5, which has somewhat more variability, the sequences of the other NTs—NGF, BDNF, and NT-3—are unusually preserved across mammals. This preservation indicates that NTs probably arose from a common ancestral gene and that their role has been conserved throughout evolution due to the crucial roles they have in nervous system development [20].

Moreover, the evolutionary conservation of NTs and their receptors across species emphasizes their importance in regulating an extensive range of cellular processes, from the differentiation and survival of nerve cells during development to the preservation of synaptic plasticity in the adult brain [21].

NTs exert their biological effects by binding to two distinct kinds of receptors on the surface of target cells: the Tropomyosin Receptor Kinase (Trk) family and the p75 neurotrophin receptor (p75NTR) [22]. These receptors are differentially expressed in various neuronal and non-neuronal tissues, and their activation facilitates the initiation of diverse intracellular signaling paths. The Trk receptors, which include TrkA, TrkB, and TrkC, are receptor tyrosine kinases that, upon binding their respective NTs, undertake autophosphorylation on specific tyrosine residues. This phosphorylation stimulates the activation of several downstream signaling cascades, including the Ras-MAPK, PI3K-Akt, and PLCγ pathways, which have a role in promoting synaptic plasticity, neuronal differentiation, and cell survival [23,24,25].

Specifically, NGF binds to and activates TrkA, BDNF, and NT-4/5 primarily bind to TrkB, and NT-3 mostly triggers TrkC, though NT-3 can also bind to TrkA and TrkB under particular conditions [26]. This discriminating but overlapping binding affinity permits a crucial regulation of NT signaling according to specific cellular contexts.

In contrast to the Trk receptors, the p75NTR belongs to the tumor necrosis factor (TNF) receptor superfamily and lacks intrinsic tyrosine kinase action. Instead, p75NTR may interact with a selection of cytoplasmic adaptors to originate signaling pathways that are greatly context-dependent [27,28]. While p75NTR can endorse cell survival in definite conditions, it has also been involved in regulating apoptotic cell death, principally in the absence of Trk receptor coactivation [24,29,30]. The pro-apoptotic signaling caused by p75NTR involves the activation of several crucial biomolecules, including caspases and members of the Bcl-2 family, such as Bax, Bad, and Bcl-xL, which eventually lead to programmed cell death in neurons [31,32,33]. Thus, the relationship between Trk and p75NTR receptors offers a subtly tuned regulatory system for defining whether a neuron could survive and differentiate or suffer apoptosis, depending on the NT accessibility and the precise cellular environment.

The dichotomy of NTs signaling—eliciting survival through Trk receptors while potentially promoting apoptosis through p75NTR—holds thoughtful implications for both nervous system development and its response to disease or injury. This subtle balance becomes mainly pertinent in pathological conditions, such as viral infections and neurodegenerative diseases, where dysregulation of NT signaling can participate in neuronal damage and/or cell death. Indeed, the role of NTs in response to infectious disorders, such as COVID-19, has collected mounting attention, as evidence indicates that SARS-CoV-2, the virus responsible for COVID-19, can provide direct and indirect actions on the nervous system. Thus, the comprehension of how NT trails are regulated in the context of viral disorders may offer precious insights into the mechanisms underlying COVID-19-related neurobiological outcomes and might indicate new possibilities for innovative therapeutic interventions.

## 2. Materials and Methods

Between May and October 2024, for this narrative review, a wide literature exploration was carried out to disclose relevant articles (Pubmed, Scopus, and WOS). The papers were chosen including the following keywords: “NGF”, “BDNF”, “COVID-19”, “SARS-CoV-2”, “Trk”, and “Long-COVID-19” (time frame 2020–2024 for COVID-19 and SARS-CoV-2). We did not filter the year of publication for the other keywords. The chosen papers were meticulously reviewed and assessed by all authors to disclose studies that potentially encounter the aim of this narrative review.

Key inclusion criteria were: (1) original studies that dealt with NGF and/or BDNF in COVID-19, (2) articles in English. Editorials, case reports, and letters were excluded from this paper. Any divergences between the authors of this study were resolved through a consensus approach.

## 3. NGF and BDNF Biological Functions in the Nervous System

### 3.1. Embryonic and Early Postnatal Development

The NGF and BDNF systems have a key role in both the maintenance and development of the nervous system. During embryonic and early postnatal development, NGF and BDNF control several crucial activities that guarantee the appropriate formation of the nervous system, including phenotypic differentiation, migration, neuronal survival, and the growth of dendrites and axons [34,35]. They also enable synapse formation, determining the intricate neural trails that are essential for cognitive, motor, and sensory functions [36,37,38]. NGF and BDNF pilot nerve cells to their proper targets, warranting that they obtain the trophic sustenance necessary for survival during critical developmental frames. In NGF and BDNF absence, nerve cells undergo programmed cell death, or apoptosis, causing disrupted functionality and development of the nervous system [39].

### 3.2. Full Postnatal Development

In the developed, fully grown nervous system, NGF and BDNF transition from supporting growth to controlling synaptic plasticity, which is the capability of synapses to weaken or strengthen over time in response to activity processes. Synaptic plasticity is essential for several higher-order brain activities, including behavioral adaptation, memory, and learning [40,41]. NGF and BDNF, particularly BDNF, are crucially involved in regulating long-term potentiation, a process by which synaptic networks become greater with reiterated stimulation, creating the biological basis for memory and learning consolidation [42,43]. Throughout their actions on synaptic plasticity, NGF and BDNF also act on behavior and mood regulation, with NGF and BDNF changes being linked to numerous neuropsychiatric disorders [44]. Outside the nervous system, NGF and BDNF have been associated with a wide range of physiological activities, including cardiovascular health [45,46,47], addiction [48,49], endocrine functions [50], immune regulation [51,52,53], and metabolic balance [54]. A huge plethora of data have confirmed that NGF and BDNF dysregulation is associated with a broad array of syndromes, including immune system dysfunctions, stress-related disorders, toxicological effects, oncological conditions, endocrine pathologies, metabolic disorders, cardiovascular diseases, and neurodegenerative syndromes [37,55,56]. For example, in neurodegenerative conditions such as Parkinson’s and Alzheimer’s disorders, significantly decreased BDNF has been disclosed, which may contribute to the advancement of cognitive decline and neuronal loss [57,58,59,60]. Likewise, psychiatric disorders like eating disorders, schizophrenia, and major depression have also been related to disrupted BDNF levels, indicating that BDNF dysfunction may be a common trail underlying different mental health situations [57,58,59,60].

## 4. NGF and BDNF Beyond the Nervous System

Although NGF and BDNF were initially reviewed in the context of their action on nerve cells, increasing pieces of evidence have potentiated their functional implication for a plethora of non-neuronal tissues. Indeed, BDNF is highly expressed in both the peripheral and central nervous systems, but its presence is not limited to these regions. BDNF is also found in several other tissues, including the thymus, heart, liver, skeletal muscle, lungs, spleen, and vascular smooth muscle cells [61,62,63,64]. This extensive expression indicates that BDNF, as well as NGF, may play various physiological tasks beyond its traditional neurotrophic functions. For example, BDNF has been shown to contribute to cardiovascular health by regulating vascular homeostasis and endothelial cell function. Furthermore, BDNF is produced by vascular endothelial cells [65], which stand on the internal surface of blood vessels, and by platelets [66] and leukocytes [67], acting in processes associated with immune response, inflammation, and wound healing. BDNF also regulates metabolism and energy balance, with emerging studies suggesting that BDNF has a key role in controlling glucose homeostasis and body weight, with action also to metabolic disruptions such as diabetes and obesity [68].

The functional diversity of neurotrophins extends to NGF, which, like BDNF, has important effects on non-neuronal cells, mostly in the immune system. The detection of NGF’s effect on immune function dates back to 1977, when it was disclosed that NGF administration in newborn rats caused a noticeable elevation in mast cells across various tissues [69]. Mast cells are immune cells located largely in connective tissues and mucous membranes, where they have an essential role in inflammatory and allergic responses [70,71]. Mast cells contain granules filled with several bioactive constituents secreted during degranulation in response to specific stimuli, participating in the body’s defense processes [72]. Both NGF receptors—p75NTR and TrkA—have been confined to the plasma membrane mast cells [73], postulating a direct indication of NGF’s action on these cells.

Successive in vitro findings have further shown that NGF can elicit mast cell degranulation, activating the release of a plethora of chemical mediators, such as cytokines and histamine, which are fundamental for inflammatory processes [73]. The occurrence of NGF receptors is not limited to mast cells; NGF receptors have also been disclosed in other main immune cells, including macrophages, monocytes, basophils, neutrophils, and eosinophils [73,74]. Throughout the interaction of NGF with these immune cells, NGF induces a chemotactic action, regulating the migration of monocytes and granulocytes to sites of injury or infection, where they contribute to phagocytosis and/or the release of pro-inflammatory biomolecules [73]. This chemotactic action is a serious feature of the innate immune response, serving to recruit immune cells to tissues where they are required to counteract pathogens and promote tissue repair.

B and T lymphocytes, the key cells involved in the adaptive immune response, also possess NGF receptors. Indeed, NGF plays a straight action in regulating lymphocyte physiology by endorsing the proliferation of T lymphocytes and boosting the differentiation of B cells into plasma cells, which have a central role in the production and secretion of antibodies [74,75]. This indicates that NGF not only stimulates early innate immune physiology but also subtly shapes the more focused adaptive immune system. Additionally, it has been noticed that some immune cells, including lymphocytes, eosinophils, and mast cells, are able to release NGF themselves, generating a feedback loop in which immune cells both contribute to and respond to the presence of NGF in their setting [76,77]. This significant capability to produce NGF focuses on the importance of NGF as a regulatory biomolecule within the immune system, where NGF coordinates the behaviors of many cell types during the immune responses.

## 5. Clinical Implications of the Neurotrophin Signaling in Infectious Disorders

In microbial infections such as sepsis, NGF and BDNF have been implicated in both protective and deleterious functions. Indeed, potentiated NGF levels have been shown in sepsis, where it seems to endorse the survival of nerve and immune cells. However, chronic overactivation of NGF and BDNF signaling could aggravate systemic inflammation, contributing to the pathogenesis of septic shock. Furthermore, p75NTR, when upregulated, may stimulate apoptosis of immune cells, thus damaging the immune response and weakening outcomes in bacteriological infections.

NGF and BDNF signaling may also play a role in viral infections [78,79,80], particularly human immunodeficiency virus (HIV) [78,81,82] and herpes simplex virus (HSV) [83,84]. These viruses often misuse NT receptors to augment their entrance into host cells or to stimulate viral persistence. HIV uses p75NTR to infect microglia and macrophages, participating in the neurodegeneration observed in HIV-associated neurocognitive conditions [85,86]. Moreover, some viruses could steal BDNF/TrkB signaling to endorse neuronal survival, permitting the virus to determine a hidden infection in the nervous system [87].

Thus, targeting NGF and BDNF signaling trails could offer putative therapeutic strategies in infectious conditions. For example, modulating Trk receptor action could decrease neuroinflammation in viral conditions to limit neural damage. Inhibiting p75NTR might be helpful in preventing the paroxysmal apoptosis of immune cells during hazardous infections. Moreover, boosting NGF and BDNF levels may have neuroprotective actions in infections with high neuroinflammatory mechanisms, such as HIV-associated neurocognitive disorders.

## 6. COVID-19 Pandemic from 2019 to Date: A Brief Overview

The COVID-19 pandemic spread globally starting in December 2019, subsequently declared a global health emergency by the World Health Organization from 30 January 2020 to 5 May 2023, and referred to as “new coronavirus disease”, more properly known by the acronym for COVID-19. The first known cases mainly involved wet market workers in Wuhan, China, where fish and other animals, including live, were sold. In the first weeks of January 2020 [88], scientists identified strange cases of pneumonia caused by a new coronavirus in these subjects, designated as SARS-CoV-2 (severe acute respiratory syndrome coronavirus 2), whose gene sequence was found to be 70% identical to that of the SARS-CoV virus, which spread with the SARS-CoV-2 epidemic of 2002–2004 [89].

The spread of SARS-CoV-2 across the globe has led, over time, to the appearance of mutations and, consequently, variants of the WIV04/2019 sequence, the zero/original sequence that developed the original form of COVID-19. Over time, the Omicron variant, along with all its subvariants, has become the prevalent variant worldwide [90].

Throughout this period, the onset of the disease was characterized by rather similar and flu-like symptoms such as dermatitis, fever, dry cough, tiredness, and breathing difficulties. In the most serious cases, often found in subjects already burdened by previous pathologies or, in the past, associated with the first two variants of the virus, pneumonia, acute respiratory and acute renal failure, and even death can develop [91,92]. Patients also present with leukopenia and lymphocytopenia.

Based on the data collected during the various studies, experts believe that the Omicron variant prefers to attack the epithelium lining the mucous membranes of the upper airways rather than the lung epithelial cells; this is evidence that contrasts with what was observed for the Delta variant of SARS-CoV-2 and the previous ones. The lower affinity for the lungs and the preference for the upper airways would seem to be the plausible reasons why Omicron infection would less often result in serious clinical pictures requiring intensive care or, in any case, hospitalization [93,94].

Certainly, one of the peculiar characteristics of SARS-CoV-2 infection is the so-called “Long-COVID-19”, a term coined to indicate the set of disorders and clinical manifestations that remain after recovery from the SARS-CoV-2 infection. For some, Long-COVID-19 may resemble the symptomatic picture experienced in COVID-19 (fatigue, breathing problems, anosmia, ageusia). For others, it may lead to new symptoms, such as problems associated with cognition (brain fog, accompanied by concentrating or memory difficulty) or with emotional disorders (anxiety and depression) [95].

The global COVID-19 pandemic has had significant consequences on the mental health of people around the world. Restrictions, social isolation, fear of the virus, and economic uncertainty have created a stressful environment that has had a profound impact on people’s psychological and emotional health.

Among the most common psychiatric symptoms recorded in the pandemic period and the period immediately following it, we can certainly include the increase in anxiety, depression, and stress, linked not only to problems relating to personal health but also to the negative socio-economic consequences. Furthermore, the lack of meaningful social interactions due to isolation policies to limit the spread of the infection has significantly favored the increase in cases of depression.

The infection due to SARS-CoV-2 alters COVID-19 host cells binding to angiotensin-converting enzyme-2 (ACE2), the SARS-CoV-2 receptor [96,97]. Accordingly, in the case of depleted O_2_ supply, the lungs, the brain, and the heart are disrupted. The binding between SARS-CoV-2 and ACE2 may thus lead to neuronal death and inflammation with the potential development of mood syndromes, such as anxiety and depression. Physical exercise also potentiates the ACE2 expression [98] but also NGF/BDNF [99,100]. Equally, the ACE2/Ang 1-7/Mas axis and NGF/BDNF activations by physical exercise may stimulate an antifibrotic anti- and inflammatory effect with beneficial effects on mental health, increasing PI3K, BDNF, IGF-1, ERK, NGF, and dropping GSK3β levels [98].

**Figure 1:**
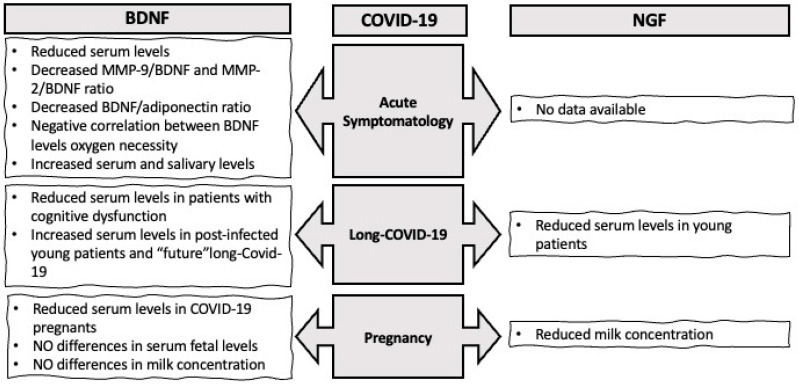
Schematic overview displaying the main results from current investigations on the involvement of NGF and BDNF in COVID-19 disease.

Given the multifactorial nature of the consequences of SARS-CoV-2 infection (physical and psychological) in adults and children, several studies have been interested in investigating the potential involvement of the NGF and BDNF systems in the pathology. This review aims to summarize the most recent evidence on this topic. Figure 1 schematically displays the main results from current investigations.

## 7. NGF and BDNF in Acute COVID-19

The first evidence of an alteration of the NGF and BDNF system (in particular BDNF) dates back to 2020, when a pilot study observed reduced levels of serum BDNF, particularly in patients with severe or moderate symptomatology as compared to patients with mild disease. Interestingly, the recovery after SARS-CoV-2 infection was associated with a restoration of BDNF levels [101].

Our study [102], involving COVID-19 patients during the first pandemic wave, disclosed that reduced BDNF in elderly individuals was associated with severe morbidity and fatal outcomes. We also predicted that the ratios of MMP-2/BDNF and MMP-9/BDNF could be considered early predictors of COVID-19 mortality, supposing the involvement of metalloproteinase [103] and BDNF [104,105] in the severe respiratory impairment associated with elevated mortality for COVID-19 infection. A subsequent study confirmed the predictive value of a decreased MMP-9/BDNF ratio for severe COVID-19 outcomes [106].

Another study extends previous findings, demonstrating that BDNF serum levels were decreased in COVID-19 patients and in those presenting neurological disorders associated with infection-induced hypoxia. Interestingly, BDNF levels had a significant negative correlation with oxygen therapy necessity [107].

An interesting study examines whether obesity, sex, and age influence the adipose tissue endocrine response in individuals affected by viral infection during the first phase of the pandemic [108]. In this study, authors demonstrated that BDNF levels, alone or in combination with adiponectin levels (BDNF/adiponectin ratio), predicted intensive care needs and were associated with worsened outcomes. Moreover, age and sex-specific differences were found since older patients (age > 60 years) had reduced BDNF levels and BDNF/adiponectin ratio in comparison to younger ones. Moreover, in men, circulant BDNF was lower than in women [108].

In a 2021 study conducted on a prospective longitudinal multicenter cohort study at four public hospitals in Singapore, the authors found elevated levels of IL-12p70, IL-17A, IL-1β, and stem cell factor and BDNF, pro-angiogenic macrophage inflammatory protein 1β, and VEGF [109]. Furthermore, the authors found potentiated levels of platelet-derived growth factor BB and monocyte chemoattractant protein-1 [109]. This and other studies clearly demonstrate that SARS-CoV-2 infection and its association with the disrupted presence of BDNF changed the association between circulating immune cells and microglia and elevated the levels of chemokines, cytokines, and inflammasomes [110]. Furthermore, the interaction between changes in NGF or BDNF in COVID-19 highlights the modifications in signaling pathways that influence neural synaptic plasticity and remodeling throughout changes in the complement system, the expression of SIRPα, CX3CL1, and CD47 biomolecules, and disrupted matrix remodeling [110,111].

More recently, results in contrast with the previous ones disclosed an increase in BDNF serum levels in COVID-19 patients with neurological disorders accompanied by increased serum inflammation (TNF-α) and oxidative stress indexes (Malondialdehyde) [112]. In line with this research, in a monocentric prospective study, salivary and circulating levels of both NGF and BDNF were found to increase in hospitalized infected patients, concerning those in the remission phase (6 months later) [113]. The authors explained that the observed NGF and BDNF increased because of the cytokine storm activation during the acute phase of the disease [114,115] and proposed NGF and BDNF dynamic biomarkers for monitoring the disease.

The interpretation of the contrasting results (increase or decrease in serum BDNF levels) is not unambiguous and well-defined. Probably the presence, at the time of sampling, of comorbidities associated with the COVID-19 pathology significantly influences the NGF and BDNF circulating levels. Furthermore, the presence or absence of neurological symptoms in the enrolled patients may be a factor that influenced the difference in the BDNF levels observed.

## 8. NGF and BDNF and Long-COVID-19

As for the involvement of NGF and BDNF in long-lasting symptomatology linked to SARS-CoV-2 contagion, few studies have taken into consideration the cognitive impairment observed after infection.

Interesting research demonstrated an association between serum BDNF levels and cognitive decline observed in a group of patients who previously experienced mild COVID-19 disease [116]. Participants were subjected to a battery of neuropsychological tests for anxiety and depression behavior, and the results have been correlated to circulating BDNF. The authors found a reduction in BDNF in patients showing persistent cognitive dysfunction concerning health-matched controls. Moreover, a gender effect on serum levels was disclosed, with male patients more affected than female participants [116].

In an opinion article, physical exercise is proposed as a promising strategy to mitigate the negative impact of long-term syndrome on brain health [117]. In this context, NGF and BDNF come into play as mediators known to be dysregulated in conditions of severe COVID-19, where an impairment of the central nervous system was documented [118,119]. On the other hand, it is known that the mechanisms through which physical exercise positively impacts behavior and mood also involve the NGF and BDNF systems, increasing BDNF expression [120,121]. In this sense, the relationship between NGF and BDNF and COVID-19 passes through an interventional strategy (physical exercise) aimed at restoring the physiological balance of the BDNF system.

In our study, we found reduced NGF serum content in a cohort of young children who contracted the infections but were negative at the time of blood sampling (post-infected-COVID-19); BDNF levels were found to increase only in post-infected-COVID-19 symptomatic girls and in those girls who would later develop “long-COVID-19” symptoms [122]. We assumed that while reduced circulating NGF could be associated with the stress condition and emotional discomfort experienced during the pandemic period, BDNF serum increase could be considered a predictor of long-lasting effect, especially in those female patients affected by the respiratory consequences of the infection.

Other data have shown that the occurrence of new or more severe headaches due to COVID-19 elicited peripheral sensitization and microglial activation associated with serum changes in BDNF, TGF-ß1, VEGF, NGF, and CX3CL1 suggestive of long-lasting severe inflammation or immune dysregulation [111].

In another investigation, the authors showed that the β-NGF/TrkA signaling pathway was correlated to the production of anti-nucleoprotein IgG in convalescent COVID-19 individuals [123], further demonstrating the subtle connection between SARS-CoV-2, the neurotrophin pathways, and immune dysregulation.

## 9. NGF and BDNF, COVID-19, and Microglia

Microglia represent a class of immune system cells resident in the brain [124,125]. They play a subtle role in the maintenance of neurons’ health by eliminating cellular debris, supporting neuronal interactions, and regulating inflammation. Crucial to their action is their association with neurotrophic factors such as NGF and BDNF [126,127,128,129]. These neurotrophins are indispensable for the growth, plasticity, and survival of brain cells, directly regulating memory, learning, and neural healing. In a healthy brain, microglia play a key role in controlling NGF and BDNF levels by reacting to environmental stimuli and sustaining a reasonable inflammatory state [124,125,126,127,128,129]. However, in the reaction to injury or infection, microglia might overactivate, shifting from their helpful action to focusing on immune defense, which may affect NGF and BDNF availability by promoting neuroinflammation.

According to COVID-19 studies, SARS-CoV-2 may easily reach the brain, triggering extensive inflammation, raising a “cytokine storm”, and stimulating microglial activation [130,131,132]. Indeed, stimulated microglia might release pro-inflammatory cytokines that support the action against viral pathogens; however, it may also decrease the neurotrophic support by modifying the neuronal signaling, reducing NGF and BDNF presence [133,134]. This decline in NGF and BDNF may lead to outcomes such as fatigue, memory impairments, and cognitive failure, outcomes now normally observed in some long-COVID-19 individuals or in the post-acute actions of SARS-CoV-2 exposure. This long-lasting microglial activation indicated that COVID-19 might contribute to protracted neuroinflammation disrupting the neurotrophic environment of the CNS, possibly developing chronic psychiatric and neurological outcomes.

Moreover, previous findings show that changes in NGF and BDNF due to continuous microglial activation might aggravate other age-linked neurodegenerative disorders, as both BDNF and NGF are crucial to constrain neuronal degeneration [135,136,137,138,139]. The association between COVID-19, neurotrophic factor reduction, and microglia activation emphasizes continuing research in exploring other therapeutic options aimed at moderating microglia overactivation, restoring NGF and BDNF presence, and decreasing inflammation.

## 10. NGF and BDNF, COVID-19, and Pregnancy

The question of whether and how contracting the SARS-CoV-2 infection could have an impact on the outcome of the pregnancy and/or on long-term consequences for the newborn has also stimulated various research in this area. Assuming the well-known involvement of the NGF and BDNF system in both neurodevelopment and immune regulation [140,141,142], some recent works have investigated the potential modification of BDNF and NGF also in women who contracted the infection during pregnancy to identify a correlation between COVID-19 and neurological outcomes in newborns. Although most are observational studies, the resulting considerations are predominantly speculative due to the lack of long-term follow-up, leaving room for hypotheses that, to date, have not found objective confirmation.

A recent study by Kirlangic and co-workers [143] illustrated the results of a prospective study in which authors evaluated the fetal neurodevelopmental status through the quantification of circulating BDNF both in serum and umbilical cord from pregnant women hospitalized with COVID-19. Maternal serum BDNF levels were lower in the COVID-19 women group compared to the healthy ones, while no differences in fetal BDNF levels were found in both study groups. The authors hypothesized that the decrease in maternal serum BDNF could reflect, rather than a direct consequence of the infection, the increase in anxiety connected to the hypothetical negative effects of COVID-19 on the unborn child, which could even lead to a greater risk of developing post-partum depression in the parturient. The lack of difference in umbilical cord BDNF values between neonates born from COVID-19 or healthy pregnant women supports the speculation that in the absence of other risk factors (i.e., prematurity), the infection does not affect fetal neurodevelopment, thanks, among others, to the BDNF-mediated neuroprotection [144,145].

Considering the critical role of NGF and BDNF in neurodevelopment, the influence of COVID-19 on BDNF and NGF in human milk was investigated. The authors revealed that in COVID-19 patients, NGF milk concentration was lower than that of unaffected donors, while BDNF levels were unchanged between groups. This observational study evidenced a dysregulation of the NGF and BDNF systems that is also detectable in human milk. However, the impact and the consequences on neurodevelopment in neonates cannot be established due to the lack of long-term evaluation [146].

## 11. Discussion and Conclusions

In the framework of COVID-19, the global pandemic triggered by SARS-CoV-2, an elevated piece of evidence has focused on the potential connections between NGF and BDNF signaling and the extensive collection of systemic and neurological outcomes observed in exposed people. It is now well-known that COVID-19 acts on multiple tissues and organs, including the nervous system, and is associated with both long-term and acute neurological impediments. Signs such as cognitive impairments, headaches, anosmia (loss of smell), and mood disturbances are common in COVID-19-affected individuals, evidencing the possibility that NGF and BDNF may be involved in the pathophysiology of this syndrome. In particular, BDNF, which is indispensable for neurogenesis and synaptic plasticity, could have a subtle role in arbitrating the consequences of viral infection on the peripheral and central nervous systems.

The association between SARS-CoV-2 and the NGF and BDNF systems may arise through different mechanisms. Primary, SARS-CoV-2 is known to elicit a potent inflammatory response, often indicated as a “cytokine storm”, which might damage the quite mild balance of NGF and BDNF signaling between the brain and other tissues. Pro-inflammatory cytokines, such as TNF-α and IL-6, are recognized to affect NGF and BDNF production and secretion, causing disrupted levels of BDNF and NGF during the infection. This modification could develop intense consequences for nerve cell physiology, participating in the neuroinflammatory and neurodegenerative activities detected in some COVID-19 individuals. Decreased NGF and BDNF could also impair the brain’s capability to repair and restore damaged nerve cells, aggravating the neurological outcomes observed in severe COVID-19 morbidity.

Additionally, the possible long-term effects of COVID-19 on the NGF and BDNF systems are a rising area of alarm. Many individuals who recovered from the acute period of infection endure experience insistent neurological problems, communally named “long-COVID-19”. Given the important role of NTs in preserving emotional regulation, neural plasticity, and cognitive function, it is reasonable that long-lasting NTs signaling dysregulation could contribute to the continuing symptoms of long-COVID-19. Thus, an improved comprehension of how COVID-19 disrupts NGF and BDNF presence, receptor activation, and downstream signaling trails could offer significant insights into the mechanisms essential for the extension of these neurological outcomes.

In addition to their straight effects on the nervous systems, NGF and BDNF may also regulate the immune response to SARS-CoV-2. Indeed, NGF possesses well-known roles in regulating immune cell activity, including macrophages, lymphocytes, and mast cells, immune cells having a main role in the body’s defense against viral infections. Understanding the interaction of the NGF-immune system may offer new chances for therapeutic interventions aimed at counteracting the harmful inflammatory consequences of SARS-CoV-2 while protecting or repairing NGF and BDNF-mediated functions.

The therapeutic inferences of NGF and BDNF dysregulation in COVID-19 are insightful. Indeed, targeting the NGF and BDNF pathways may offer an innovative approach to curing the systemic and neurological effects of this infection. For example, approaches aimed at elevating BDNF could support and counteract the neurodegenerative and cognitive changes associated with both long-term and acute COVID-19. Similarly, the modulation of NGF physiology may regulate the disproportionate immune response observed in severe COVID-19, possibly decreasing inflammation to stimulate tissue regeneration and repair.

In conclusion, the NGF and BDNF systems, with their extensive impact on neuronal and non-neuronal cells, may have a crucial role in the COVID-19 pathophysiology and recovery. The complex association between NGF and BDNF, nerve cell health, and immune responses indicates that the NGF and BDNF system may be a significant objective for the knowledge of the neurobiological and systemic outcomes due to SARS-CoV-2 or other coronaviruses. Of course, future research studies are required to elucidate this and other mechanisms by which *(i)* NGF and BDNF participate in the various clinical expressions of COVID-19 and *(ii)* to discover how and why the NGF and BDNF signaling modulation might provide therapeutic benefits.

## Data Availability

Not applicable.
